# Digital Workflow for Indirect Bonding with 2D Lingual Brackets: A Case Report and Procedure Description

**DOI:** 10.1155/2019/6936049

**Published:** 2019-04-28

**Authors:** Federico Rosti, Maria Francesca Sfondrini, Davide Bressani, Marina Consuelo Vitale, Paola Gandini, Andrea Scribante

**Affiliations:** ^1^Private Practice, Cesano Boscone, Italy; ^2^Unit of Orthodontics and Paediatric Dentistry-Section of Dentistry-Department of Clinical, Surgical, Diagnostic and Paediatric Sciences, University of Pavia, Pavia, Italy; ^3^Dental Technician, Pavia, Italy

## Abstract

**Objective:**

During orthodontic therapy, accuracy in bonding procedures makes it easier to correct tooth alignment by decreasing the need for midcourse corrections by changing bracket positions. Indirect bonding allows the transfer of the appliance components from model casts to patient's teeth potentially meaning shorter appointments for bracket bonding and rebonding and best comfort during chairside practice. At the same time, there has been a steady increase in requests for invisible lingual orthodontic treatment.

**Clinical Considerations:**

Accordingly, the aim of the present report is to illustrate the workflow to realize a complete digital indirect bonding for lingual brackets (2D, Forestadent). The procedure starts with intraoral digital scans, digital 3D model, and virtual bracket positioning, ending with the realization of a CAD-CAM prototyped transfer tray. A 3D intraoral scanner (True Definition, 3M) is used to create digital scans and digital models. A virtual bracket positioning is performed using software (NemoCast, Dentaurum), and a prototyped transfer tray is created by a CAD-CAM device. 2D lingual brackets were positioned inside the tray, so the appliance was bonded to the dental surface using light curing adhesive resin.

**Conclusions:**

During orthodontic treatment, CAD/CAM technology could help clinicians. Computer-constructed transfer trays can reduce clinician error and improve the everyday workflow in the office.

## 1. Introduction

In straight-wire orthodontic devices, ideal bracket placement can correct tooth position in the three planes of space during treatment [1]. Accuracy in bracket placement allows an efficient orthodontic treatment [2, 3].

According to Carlson and Johnson [4], there are four elements necessary for ideal bracket placement: (1) bracket base adaptation to dental surface, (2) evaluation of the rotational bracket position in relation to the occlusal plane, (3) determination of vertical position of each bracket, and (4) determination of slot angulation according to the position of the roots.

Several studies claim that indirect bonding can be more accurate than direct bonding on labial appliances with a reduction in bracket position errors in each of the three orientations examined [5–7]. Accuracy in bracket placement can also reduce the need for repositioning and can then shorten treatment time [8, 9]. These studies were related to labial and not lingual appliances and results, however, are preliminary and inconclusive about precision of positioning and total treatment time. Therefore, the reduction of chairside time can be considered the main real advantage of indirect bracket placement.

Because of its outstanding aesthetic preconditions and its growing practicability, lingual orthodontics accounts for an ever-increasing percentage of orthodontic treatments and even more studies evaluated various aspects of this technique [10–14]. Although the lingual orthodontic treatment provides several aesthetic advantages, its use has been limited due to the increased chair time and more difficult mechanical control [15]. Placement of lingual orthodontic appliance can be made easier however with new technologies such as intraoral scanning, virtual positioning of brackets, and computer-aided transfer tray manufacturing [16, 17].

Advances in CAD/CAM technology are providing new possibilities in orthodontics: the application of CAD/CAM for establishing a virtual set-up or creating transfer trays/jig [16, 17] has improved the indirect bonding workflow.

The aim of this manuscript is to illustrate the workflow to realize a complete digital indirect bonding, after an intraoral scanning and construction of a prototyped transfer tray has been created for indirect bonding.

## 2. Case Report

### 2.1. Diagnosis and Aetiology

**Figure 1:**
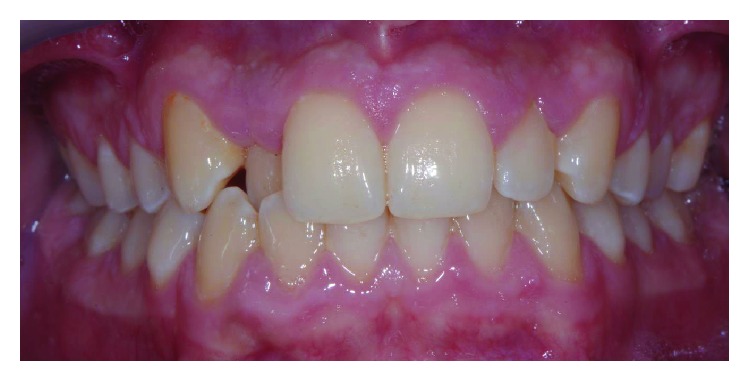
Front view of initial occlusion.

**Figure 2:**
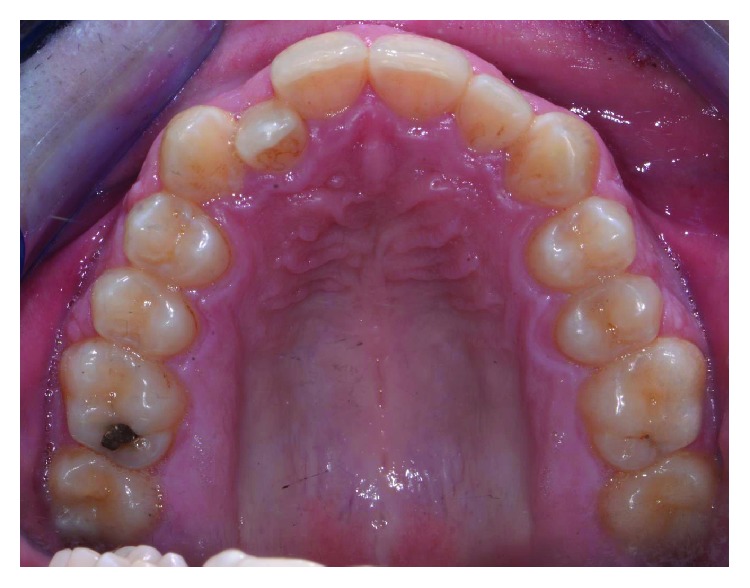
Occlusal view of initial occlusion.

**Figure 3:**
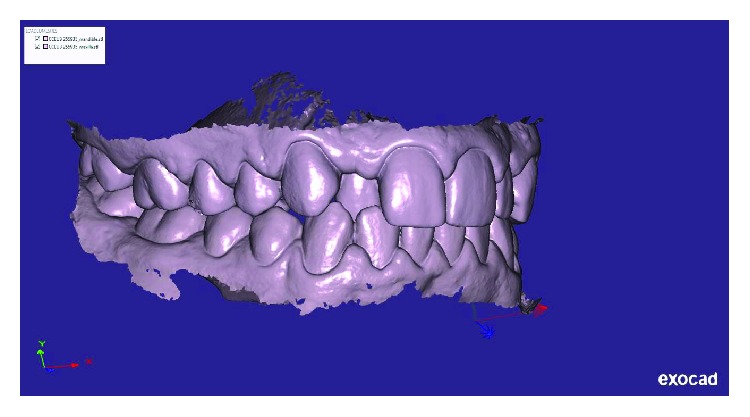
3D intraoral scan view of initial occlusion.

A male patient, aged 19 years old, was referred to a private orthodontic practice. He was diagnosed with a bilateral Class I canine relationship and left Class I molar relationship in the permanent dentition with a crossbite of tooth 12 (Figures 1–3). Patient chief complaint was to resolve the anterior crossbite with an invisible orthodontic appliance.

### 2.2. Treatment Objectives

A lingual orthodontic appliance has been projected using a CAD/CAM digital workflow. The objective of the treatment was the resolution of anterior crossbite and retention of the case over time [18].

### 2.3. Treatment Alternatives

As the patient wanted to avoid conventional vestibular orthodontic treatment, a lingual orthodontic appliance was chosen. Other alternatives to conventional visible metallic attachments would have been orthodontic treatment with ceramic brackets or aligners that are both effective to obtain tooth movement [19, 20]. The patient, however, wanted to avoid ceramic brackets because of the possibility of adverse staining midtreatment and refused aligners so as to avoid treatment with removable appliances.

### 2.4. Treatment Progress

**Figure 4:**
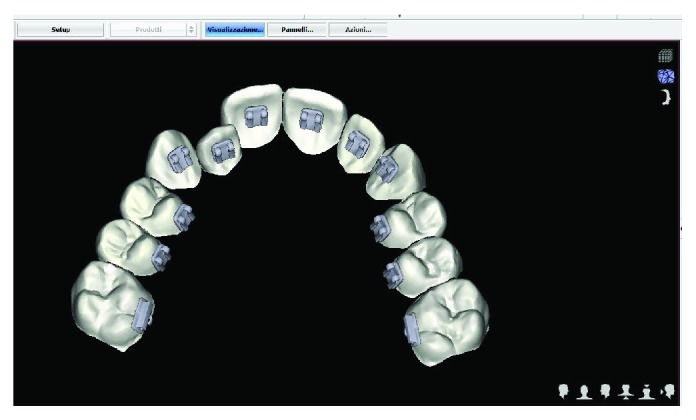
Case planning with virtual brackets.

**Figure 5:**
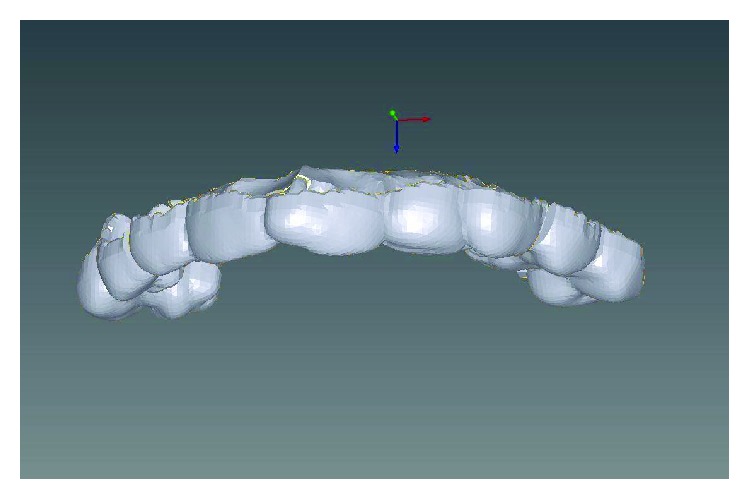
Virtual transfer tray.

**Figure 6:**
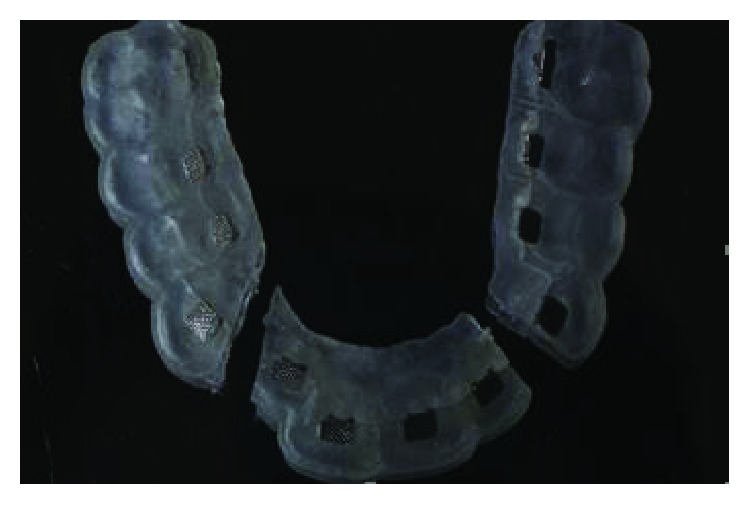
Prototype of digital transfer tray.

**Figure 7:**
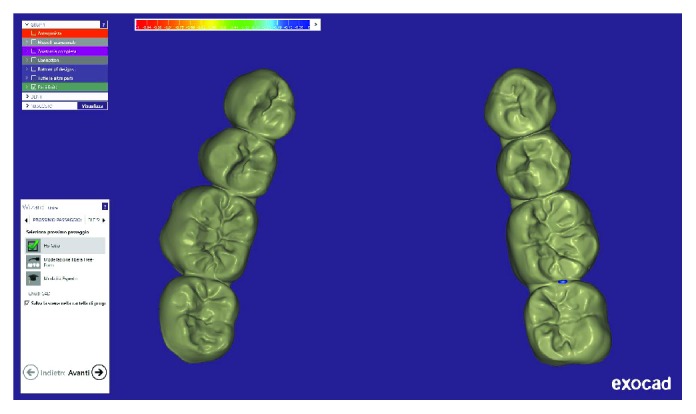
Virtual customized bite raisings.

**Figure 8:**
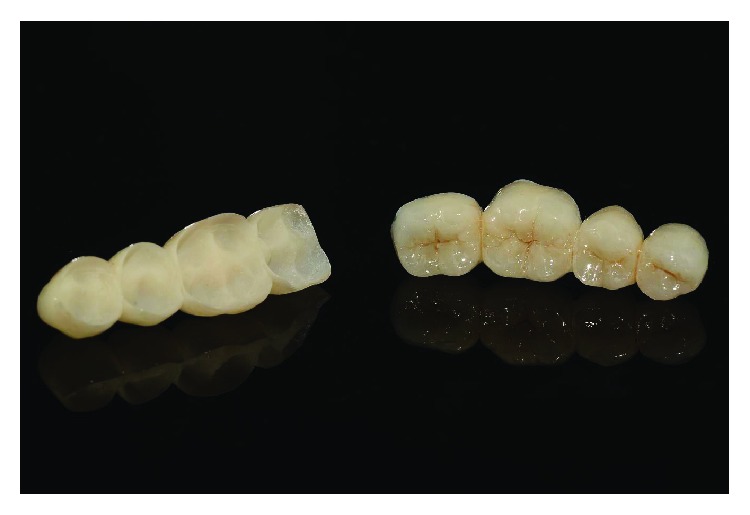
Customized bite raisings.

Written informed consent was obtained by the patient to proceed with orthodontic diagnosis and treatment. The procedure for the virtual indirect bonding started with the polishing of all dental surfaces with pumice powder; then, intraoral scan (Figure 3) was performed by a 3D intraoral scanner (True Definition, 3M, US). NemoCast software (Dentaurum, Germany) acquired the 3D models and recognized the shape of each tooth and the gum. The software realized a virtual set-up, and then, the orthodontist placed the virtual brackets on 3D virtual models according to the lingual prescription (Figure 4). The bracket placement tool ensured the precise bracket positioning according to the virtual set-up using the virtual bracket's position on the screen. Once all brackets were positioned on the 3D models, the software allowed designing a virtual transfer tray for indirect bonding (Figure 5). A prototype of the digital transfer tray was manufactured using a rapid-prototyping machine (Figure 6). exocad software (exocad GmbH, Germany) was used to create bilateral posterior overlays to act as customized bite raisers (Figures 7 and 8).

**Figure 9:**
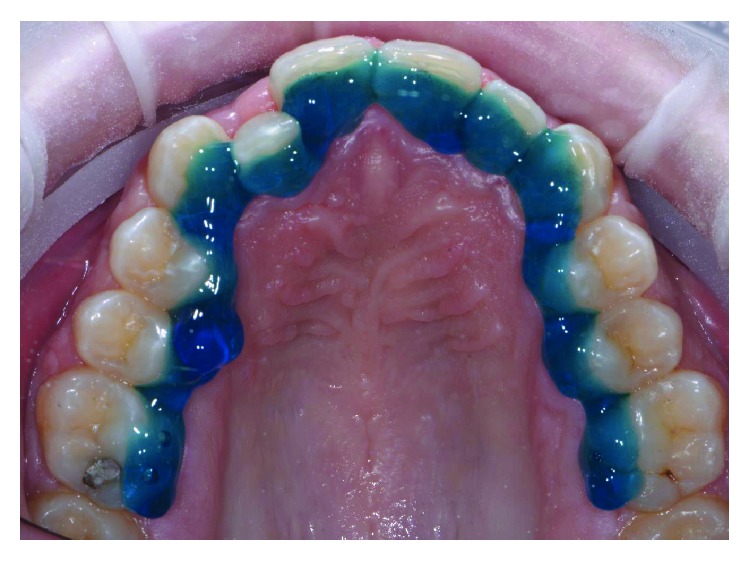
Etching procedure.

**Figure 10:**
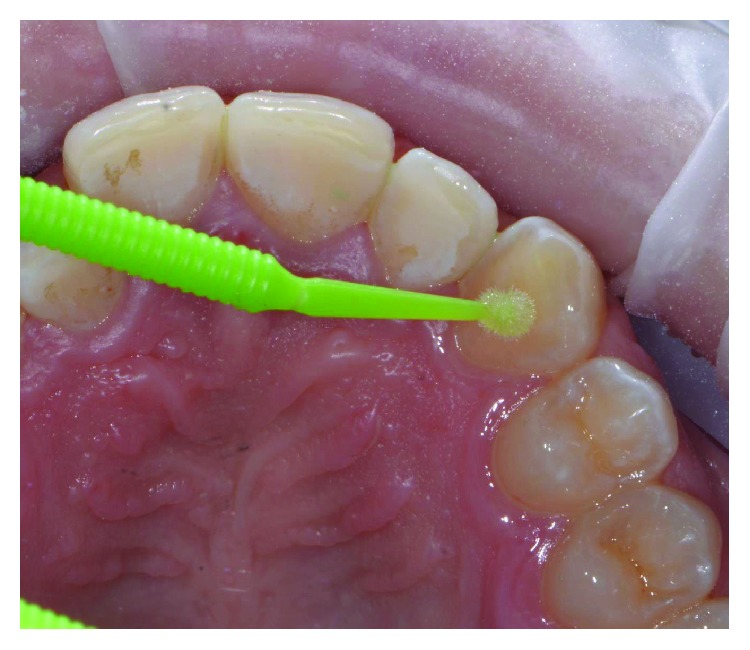
Adhesive application.

**Figure 11:**
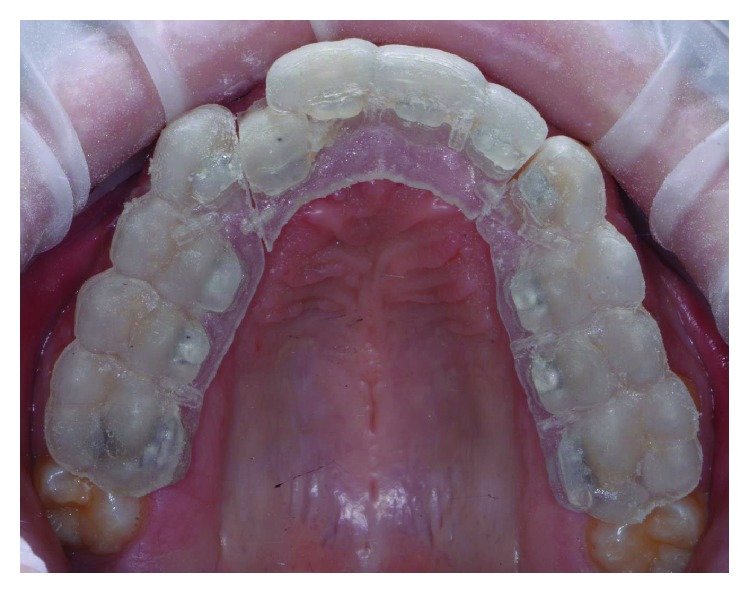
Device placement.

**Figure 12:**
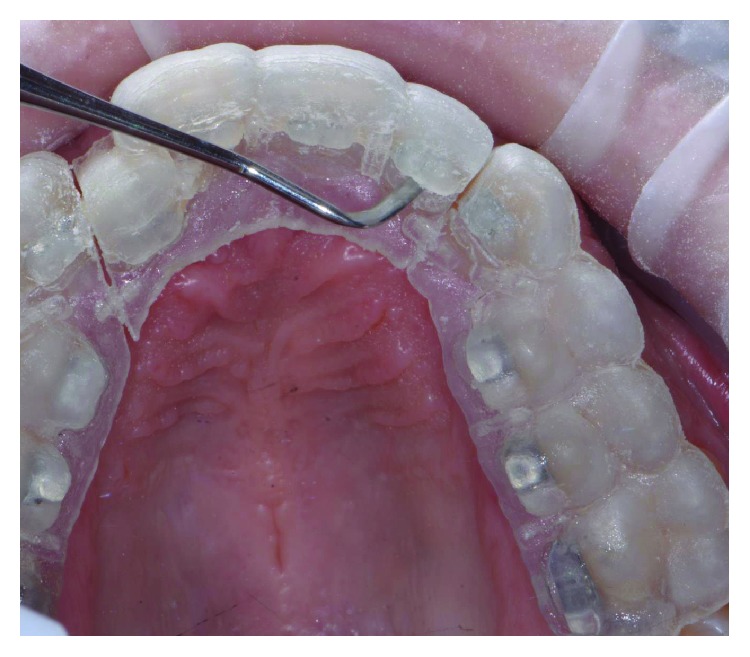
Transfer tray removal.

**Figure 13:**
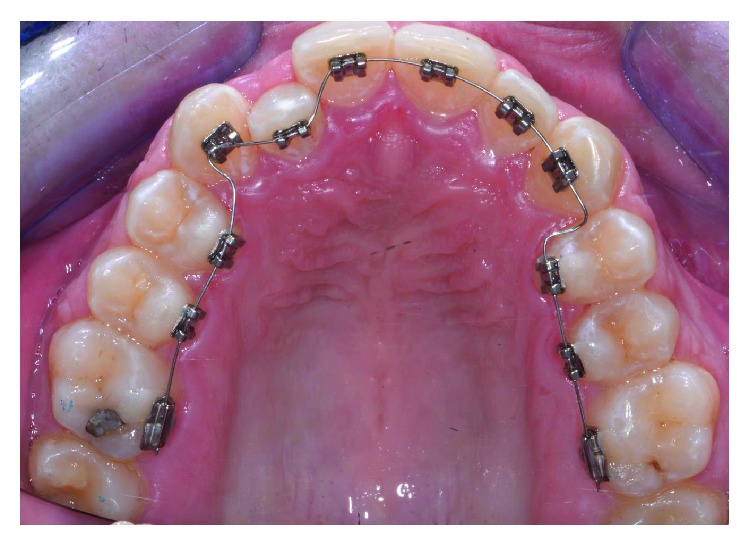
Wire placement.

The patient wore protective glasses to prevent eye damage [21], and the tooth surfaces were first etched with 37% orthophosphoric acid gel (3M, US) for 30 seconds (Figure 9) followed by washing and drying. A primer (Transbond XT, 3M, US) was applied in a thin film on the etched tooth surface (Figure 10). 2D lingual brackets (Forestadent, Germany) were positioned inside the prototyped transfer tray, and an adhesive resin (Transbond XT, 3M, US) was applied over the bracket bases [22]. The brackets were then positioned in the maxilla on the upper teeth (Figure 11), using the prototyped transfer jig, and were light cured for 60 seconds each by an LED lamp (Elipar DeepCure, 3M, US). Subsequently, the transfer tray was removed by forcing a probe in the fracture line (Figures 12 and 13) and separating the jig in two halves. The fracture line was created by the CAD software in order to be useful for an easy transfer tray removal.

**Figure 14:**
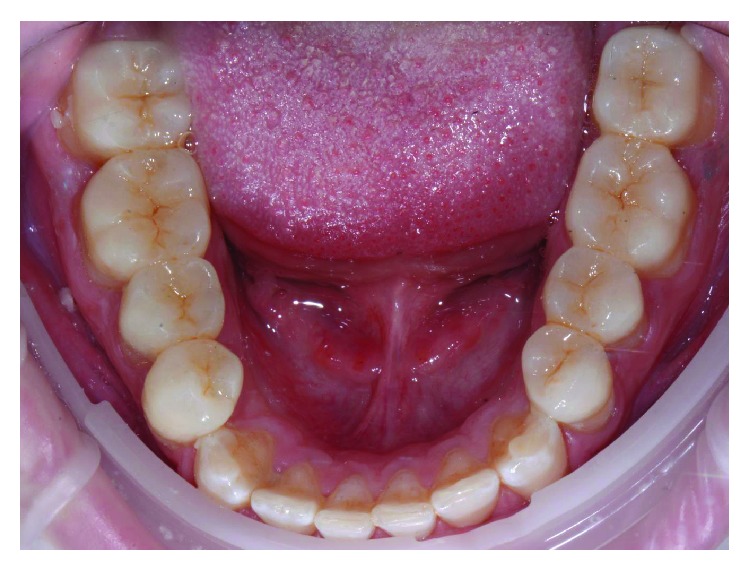
Setting of prototyped overlays.

**Figure 15:**
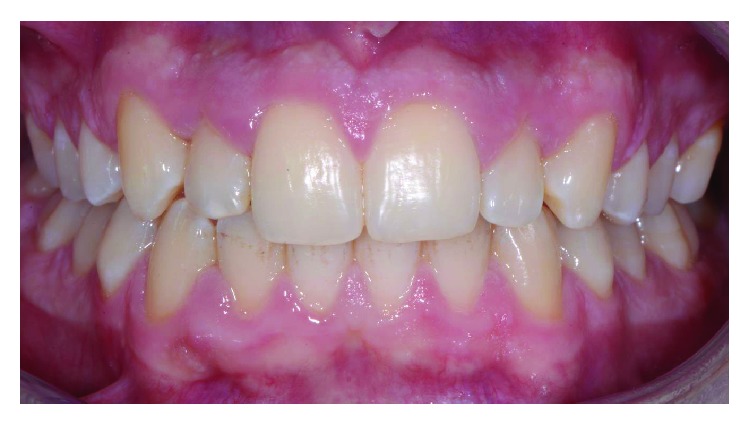
Front view of final occlusion.

**Figure 16:**
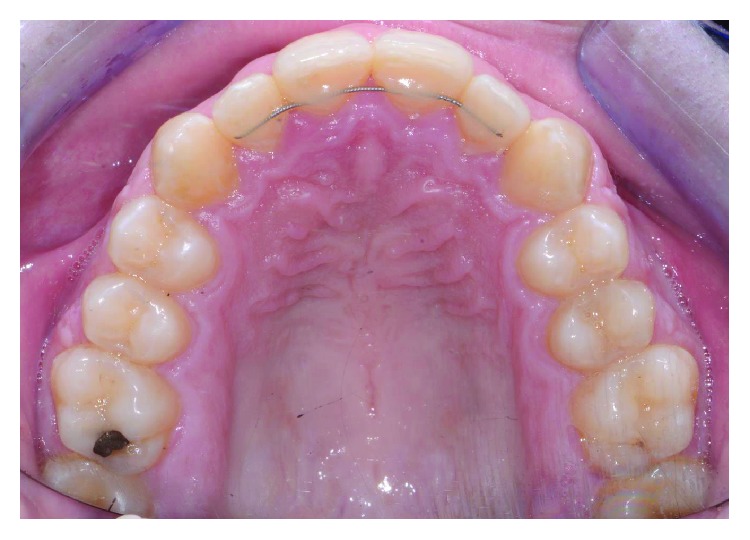
Occlusal view of final occlusion.

On the lower jaw, prototyped overlay were set using an adhesive technique (Figure 14). Orthodontic treatment progress included 0.012 inch, 0.014 inch, and 0.016 inch nickel titanium archwires, followed by 0.16 stainless steel wire. The patient was checked each month, and wires were changed after 2 months each. Finally, brackets were removed, and teeth were polished (Figures 15 and 16). An upper splint and an upper Essix removable appliance were placed in order to guarantee posttreatment stabilization [23, 24].

## 3. Discussion

The virtual indirect bonding has been performed with lingual brackets with the treatment plan involving the upper teeth alone by resolution of the lateral incisor crossbite. The case has been planned with a digitalized workflow with CAD/CAM digital impression taking presenting similar efficiency as traditional impression methods in orthodontics and being more comfortable for patients [25, 26]. The position of brackets on the patient teeth (Figure 13) corresponded to the position of the same brackets on the digital 3D models (Figure 4). This can be obtained as CAD/CAM technology shows a variety of possibilities in orthodontics from study design [25] to the construction of customized splints [15] and trays for vestibular appliances [27] or for maxillofacial surgery scopes [28]. To our knowledge in the literature, there are no studies that evaluated CAD/CAM preparation of transfer trays for standard lingual appliances realized by an independent dental technical laboratory. In fact, the CAD/CAM transfer tray technique is performed only directly from the manufacturer for the use of customized lingual brackets [29], which present higher realization costs than standard lingual appliances using this technique [30].

Accurate surface imaging is required to digitally manufacture orthodontic appliances with 3D intraoral scanners reproducing precisely the image of the lingual dental surfaces [31]. Lingual surfaces vary more widely than labial surfaces [32]: for example, the bicuspid has a very slim and irregular lingual surface making bracket placement difficult. An advantage of the CAD design method is improved bonding of the lingual bracket base [33], which is important for adhesion performance [34]. The software can realize customized bracket bases which are useful in the case of bicuspids with very small lingual surfaces. Finally, the highest resolution of commercially available stereolithographic printers is about 0.3 mm, which is sufficient for providing both the bracket retention inside the prototyped transfer tray and the retention feature on the base of a stereolithographic prototype [35].

The computerized tool provides accuracy in bracket placement and significant reduction in chairside time. While software can provide precise reproducibility, orthodontists can incorporate alterations from the ideal placement to provide overcorrection of rotated teeth or accentuated tip to resist root motion in space closure [15]. Virtual indirect bonding can also be exported to other 3D CAD software, e.g., to realize customized occlusal bite raisers. Using the virtual articulation software, premature contacts on the brackets can also be eliminated.

Indirect bonding is currently used with 3D customized lingual brackets, and it is not used with the 2D lingual appliance, as the brackets can be placed directly. Moreover, 2D lingual brackets are a low cost option for mild cases where only the 1st- and 2nd-order corrections are required as there is no prescription in the bracket for torque correction (no 3rd-order modifications are possible). However, the present report evaluated indirect bonding for the 2D lingual system in order to reduce chair time of the bracket positioning appointment. Moreover, during direct bonding procedure on the lingual side of the tooth, often the clinician does not have a clear vision of the bonding area. Therefore, the advantage of a CAD/CAM indirect bonding is the possibility to zoom on details and provide a better position of the lingual bracket on the lingual surface. The implementation of a simplified lingual technique with CAD/CAM technology could offer orthodontic clinicians new interesting and feasible possibilities.

## 4. Conclusions

After an intraoral scan, a digital indirect bonding technique has been shown to be possible using the standard lingual brackets which is both repeatable and effective.
